# Supplementary material for: Sex Differences of Radiation Damage in High-Fat-Diet-Fed Mice and the Regulatory Effect of Melatonin
Source: Nutrients. 2022 Dec 23;15(1):64. doi: 10.3390/nu15010064 (PMC9823527; doi:10.3390/nu15010064)
Supplement: Supplementary file 1 [file nutrients-15-00064-s001.zip › nutrients-2059133-supplementary.pdf]

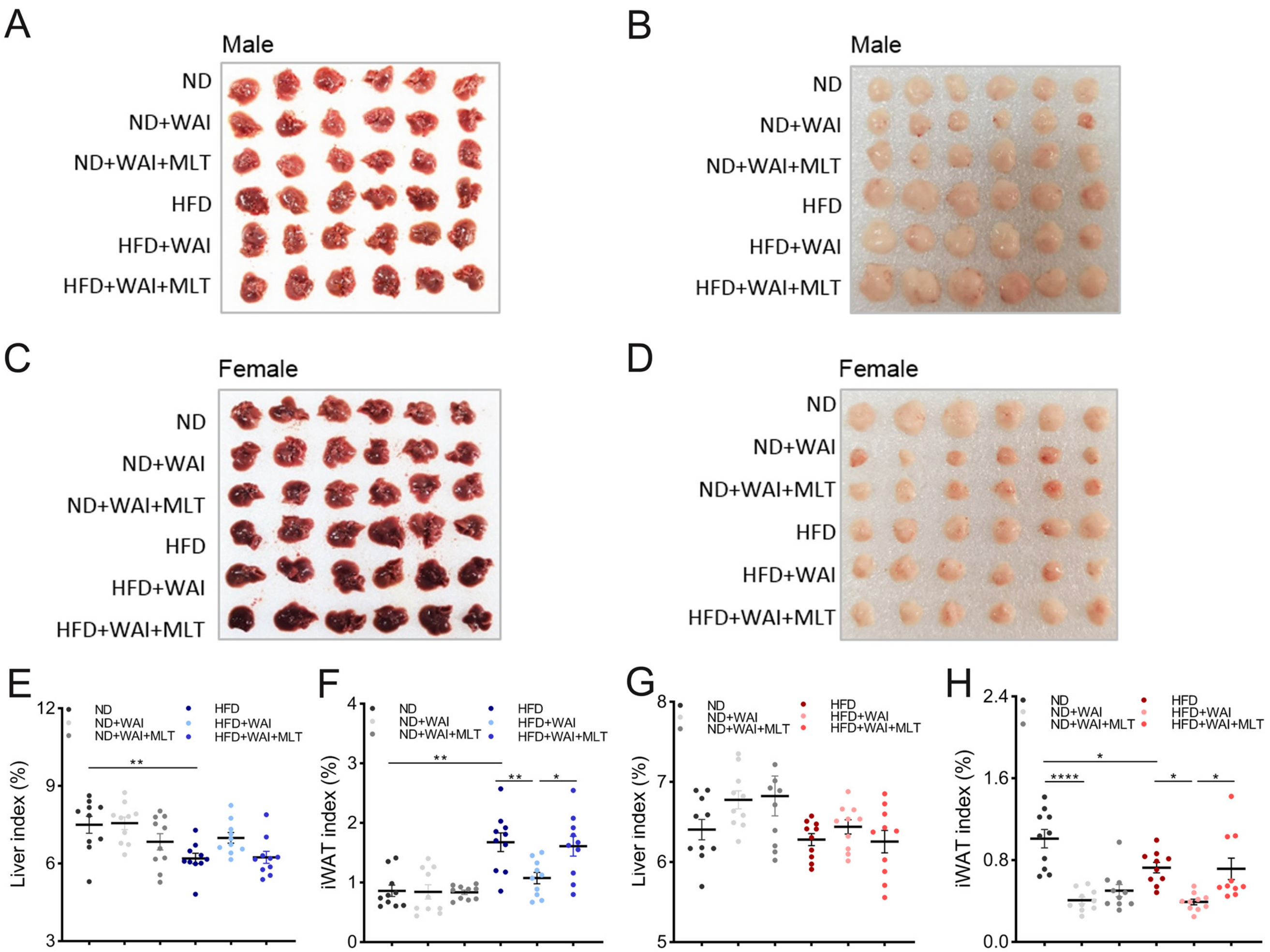

Figure S1. Photographs of the liver and iWAT from male (A, B) and female (C, D) mice in the six groups. The liver index (E) and iWAT index (F) of the male mice are shown. The liver index (G) and iWAT index (H) of the female mice are shown. Liver index = liver weight/body weight. iWAT index = iWAT weight/body weight. The results are shown as the mean ± SEM, **p* < 0.05, ***p* < 0.01, *****p* < 0.0001 between the two cohorts (*n* = 10 mice per group). (MLT, melatonin; iWAT, inguinal white adipose tissue; WAI, whole abdominal irradiation; SEM, standard error of mean).

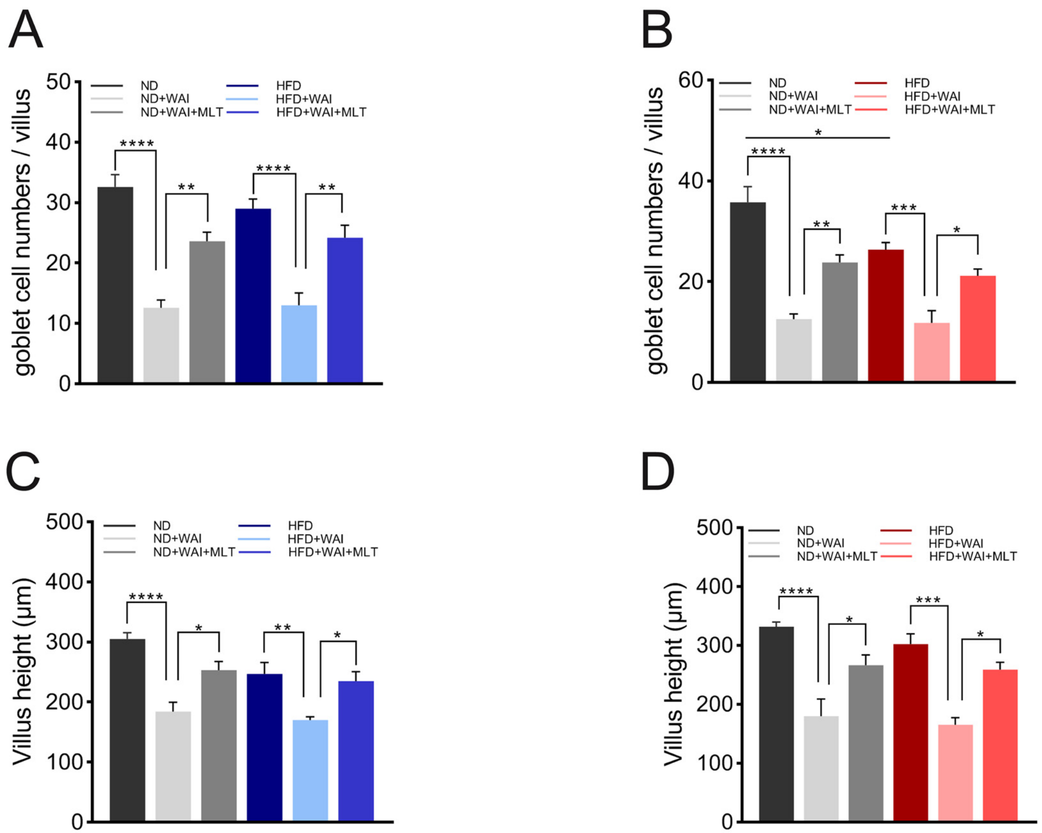

Figure S2. MLT promotes small intestinal structure repair in mice following WAI. The number of goblet cells in the villi was quantified in male (A) and female (B) mice. Quantification of villus height in male (C) and female (D) mice in intestinal sections. Data are presented as mean ± SEM, *$p < 0.05$, **$p < 0.01$, ***$p < 0.001$, ****$p < 0.0001$ between the two cohorts (5 views per group). (MLT, melatonin; WAI, whole abdominal irradiation; SEM, standard error of mean).

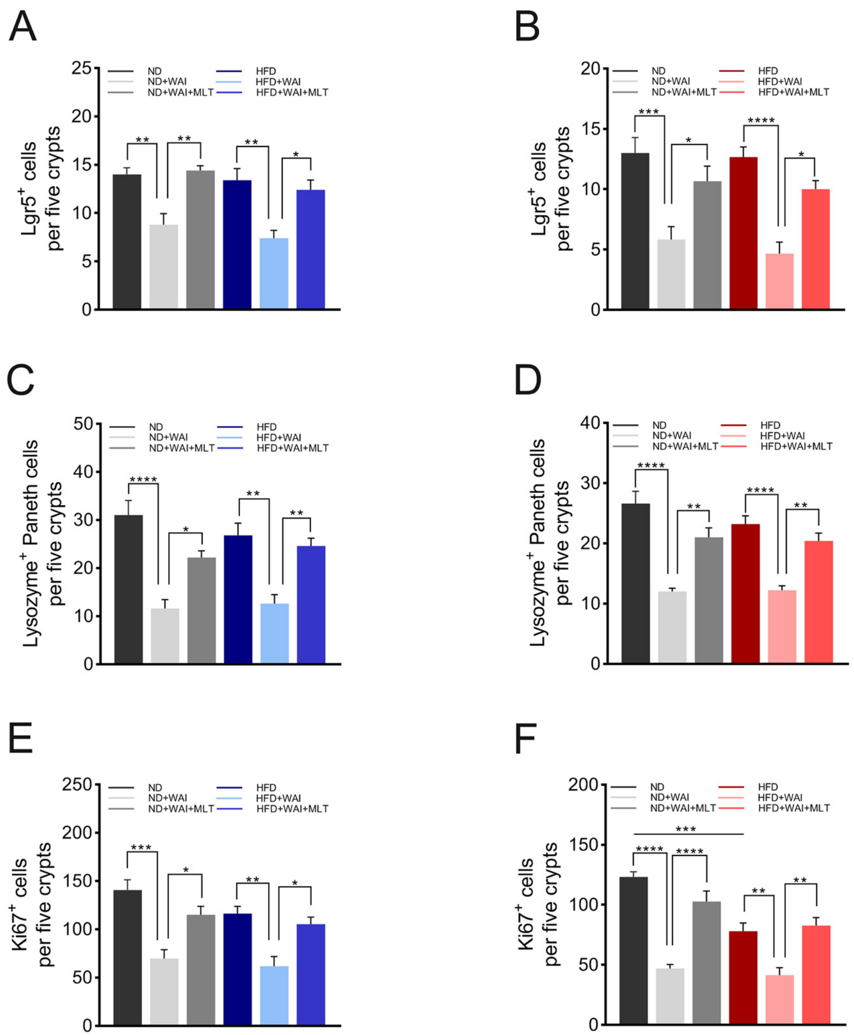

Figure S3. MLT enhances the regenerative ability of Lgr5+ ISCs after WAI. The numbers of Lgr5+ (A), lysozyme+ (B), and Ki67+ (C) cells per image were quantified. The results are shown as the mean ± SEM, **p* < 0.05, ***p* < 0.01, ****p* < 0.001, *****p* < 0.0001 between the two cohorts (5 views per group). (MLT, melatonin; WAI, whole abdominal irradiation; SEM, standard error of mean).

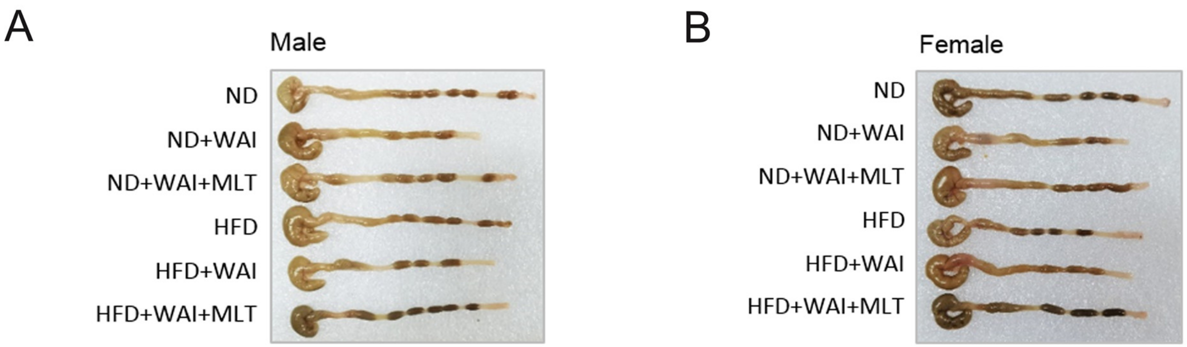

Figure S4. MLT alleviates WAI-induced colon injury. Mice were treated with an ND or HFD for two weeks. They were then irradiated with 15 Gy WAI after intraperitoneal injection of MLT. Images of the colon from male (A) and female (B) mice in the six groups. (MLT, melatonin; WAI, whole abdominal irradiation; HFD, high-fat diet; ND, normal diet).

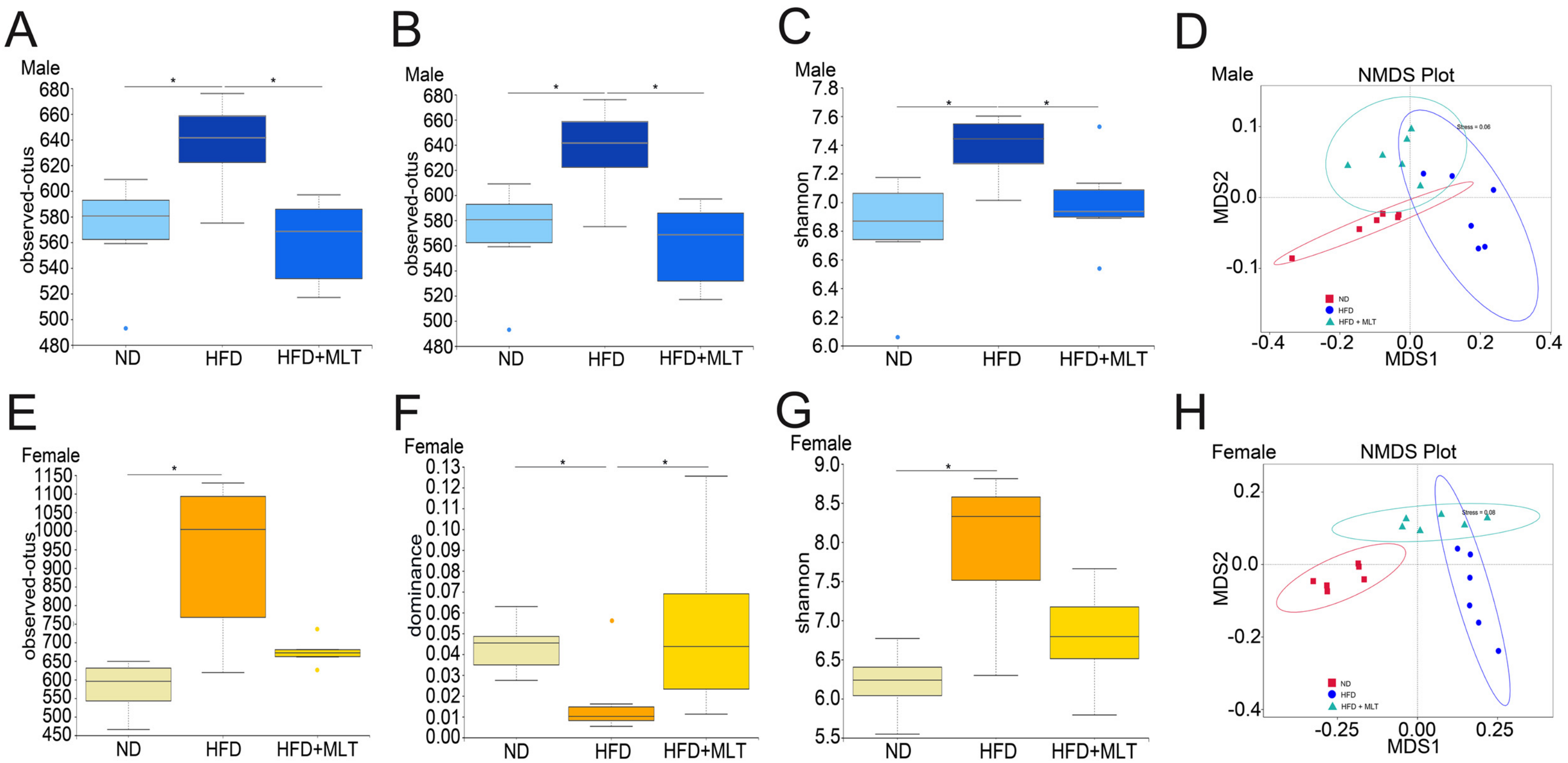

Figure S5. MLT treatment restores the changes in gut microbiota abundance and diversity caused by HFD. After treating the mice with an HFD and MLT for two weeks, feces were collected and evaluated by 16S rRNA high-throughput sequencing. The observed OTUs, dominance index, and Shannon index of enteric bacteria were measured in male (A-C) and female (E-G) mice. Non-metric multidimensional scaling (NMDS) was used to assess the gut microbiome taxonomic profiles of male (D) and female (H) mice (n = 6 mice per group). The results are shown as the mean ± SEM, *$p < 0.05$ between the two cohorts. (HFD, high-fat diet; MLT, melatonin; OTUs, operational taxonomic unit).

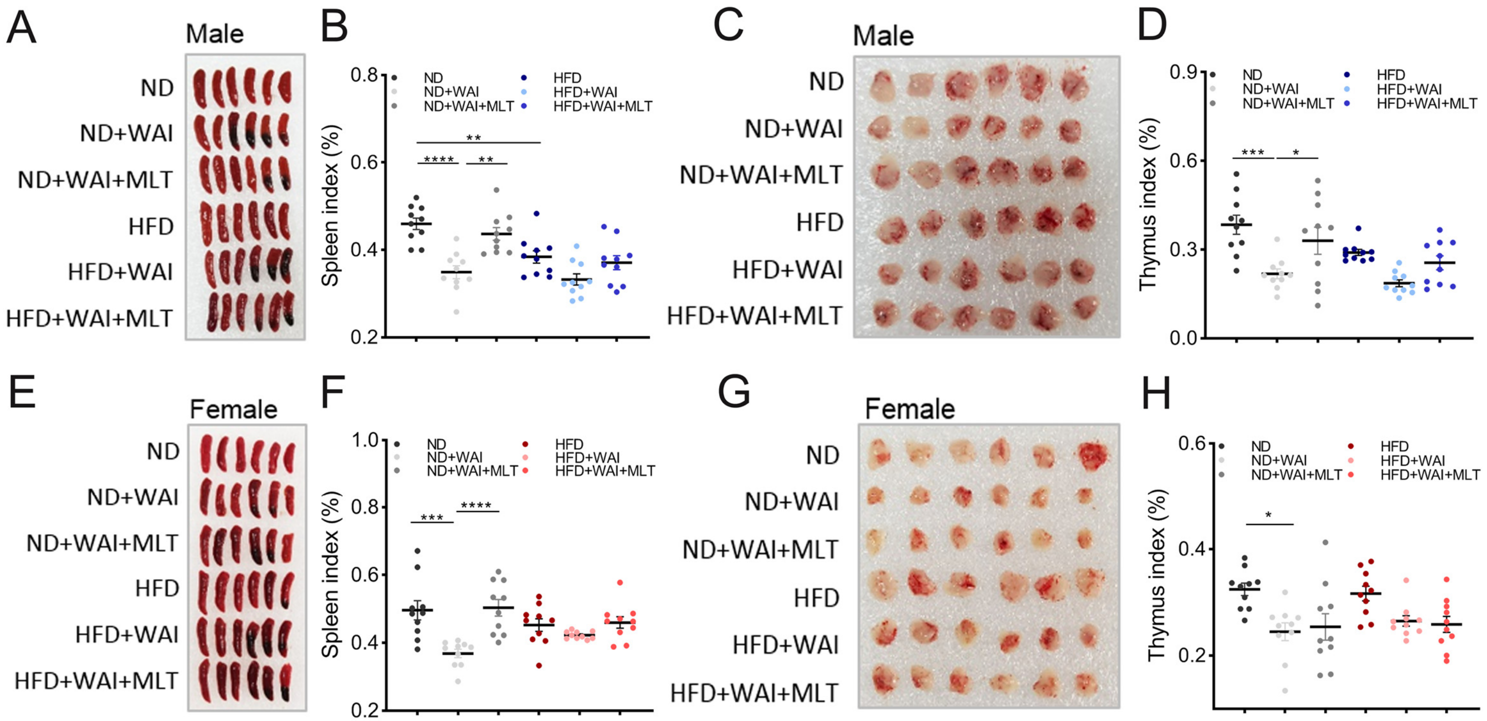

Figure S6. MLT prevents hematopoietic system injury in WAI mice. Photographs of the spleen and thymus from male (A, C) and female (E, G) mice in the six groups. Spleen index (B) and thymus index (D) of the male mice are shown. The spleen index (F) and thymus index (H) of the female mice are shown. Spleen index = spleen weight/body weight. Thymus index = thymus weight/body weight. The results are shown as the mean ± SEM, *$p < 0.05$, **$p < 0.01$, between the two cohorts (n = 10 mice per group). (MLT, melatonin; WAI, whole abdominal irradiation; SEM, standard error of mean).
